# Vibration Welding of PLA/PHBV Blend Composites with Nanocrystalline Cellulose

**DOI:** 10.3390/polym16243495

**Published:** 2024-12-15

**Authors:** Patrycja Bazan, Barbara Kozub, Arif Rochman, Mykola Melnychuk, Paulina Majewska, Krzysztof Mroczka

**Affiliations:** 1 Faculty of Materials Engineering and Physics, Cracow University of Technology, Warszawska 24, 31-155 Cracow, Poland; barbara.kozub@pk.edu.pl (B.K.); paulina.majewska@student.pk.edu.pl (P.M.); krzysztof.mroczka@pk.edu.pl (K.M.); 2 Department of Industrial and Manufacturing Engineering, University of Malta, MSD 2080 Msida, Malta; arif.rochman@um.edu.mt; 3 Department of Materials Science, Lutsk National Technical University, Lvivska 75, 43018 Lutsk, Ukraine; m.melnychuk@lntu.edu.ua

**Keywords:** PLA/PHBV blends, vibration welding, optimalization, mechanical properties, nanocristaline cellulose

## Abstract

Thermoplastic composites have garnered significant attention in various industries due to their exceptional properties, such as recyclability and ease of molding. In particular, biocomposites, which combine biopolymers with natural fibers, represent a promising alternative to petroleum-based materials, offering biodegradability and reduced environmental impact. However, there is limited knowledge regarding the efficacy of joining PLA/PHBV-based biocomposites modified with nanocrystalline cellulose (NCC) using vibration welding, which restricts their potential applications. This study demonstrates that vibration welding enables efficient bonding of PLA/PHBV composites with NCC, resulting in strong, biodegradable, and environmentally friendly materials. The investigation revealed that the addition of nanocrystalline cellulose (NCC) at 5, 10, and 15 wt.% significantly enhanced the strength of welded joints, with the highest strength achieved at 15% NCC content. Microstructural analysis using scanning electron microscopy (SEM) and deformation studies with digital image correlation (DIC) indicated that a higher NCC content led to greater local deformation, reducing the risk of brittle fracture. Mechanical hysteresis tests confirmed the composites’ favorable resistance to variable loads, highlighting their stability and energy dissipation capabilities. Optimization of welding parameters, such as vibration amplitude, welding time, and pressure, is crucial for achieving optimal mechanical performance. These findings suggest that PLA/PHBV composites modified with NCC can be utilized as durable and eco-friendly materials in various industries, including automotive and packaging. This research presents new opportunities for the development of biodegradable high-strength materials that can serve as alternatives to traditional plastics.

## 1. Introduction

Thermoplastic composites have become highly favored in numerous industries as a result of their exceptional features and benefits, which include their recyclability and moldability [1]. They have gained extensive usage, particularly within the automotive and aerospace industries, due to their attributes of lightness, strength, and feasibility of repair [2,3]. In the modern era, there is heightened interest in materials that offer efficient production methods, high resistance to damage, and design versatility. Compared to thermosets, thermoplastic composites are more desirable due to their ease of recycling and weldability, making them suitable for diverse industrial applications [4,5]. The growing concern over the consumption and price of oil has led to a heightened interest in biopolymers and biocomposites. Biocomposites, which are made by reinforcing biopolymers with natural fibers, are gaining recognition as environmentally friendly alternatives to petroleum-based polymers, particularly in the automotive industry [6]. Biocomposites provide a promising solution for various applications due to their combination of biodegradability, reduced environmental impact, economic advantages, and enhanced mechanical properties [7]. The primary objective of basic research is the production of materials with high strength and practical applications.

Several techniques are available for joining thermoplastic composites, including bonding, mechanical fastening, self-piercing riveting, laser bonding, and spot joining. Each of these methods has its own set of advantages and applications, which can impact the versatility and efficiency of joining these materials. It is important to note that there are various methods for joining thermoplastic composites, each with its own unique advantages and applications. Among these methods, adhesive bonding, mechanical fixation, and fusion bonding are considered the three primary methods [8]. Adhesive bonding and mechanical fastening are commonly employed in the assembly of metals and plastic composites [9], offering versatility and dependability. Additionally, friction welding is an emerging technique for producing plastic-to-metal joints without the use of additives, particularly in lap configurations. This method has demonstrated its potential to create strong bonds between aluminum and polyethylene, making it a promising option for various material combinations [10].

Several techniques can be utilized for welding thermoplastic composites, each with its own distinct advantages and applications. The most frequently employed method is ultrasonic welding, which employs ultrasonic vibrations to produce heat at the joint contact, resulting in the melting and bonding of materials. This technique boasts a short processing time, high strength, repeatability, and low energy consumption [11,12].

Vibratory welding is a widely recognized method for joining thermoplastic composites. This method relies on the generation of heat through friction, which is generated when two pressurized components vibrate along their mutual contact surface. The process is characterized by a number of intricate phenomena, including friction, melting, rapid deformation, pressurized melt flow, and microstructure development, all of which have an impact on the strength of the resulting weld [13,14]. This method is also characterized by its simplicity, efficiency, and ability to establish strong connections. It is well suited for applications that require solid-state joining and produces high-quality joints at a relatively low cost [15]. Vibratory welding is a process that produces strong and durable joints. It relies on the application of high-frequency, low-amplitude mechanical vibrations to create friction and heat, which then leads to the bonding of materials [16].

The implementation of vibration welding techniques in the bonding of PLA (polylactic acid) and PHBV (poly(3-hydroxybutyrate-co-3-hydroxyvalerate)) mixtures presents a promising approach to enhancing the mechanical properties and functionality of biopolymer composites by improving material flow and reducing defects at the weld interface [17].

For example, Zhao et al. [18] demonstrated that controlled processing conditions facilitate the optimization of the phase morphology and mechanical properties of PLA/PHBV mixtures, resulting in enhanced tensile strength and ductility. The application of vibration welding can further improve these properties by reducing residual stresses and enhancing the distribution of the polymer phases. The ratio of PLA to PHBV is of critical importance in this context, as increasing the proportion of PHBV alters the behavior of the mixtures from brittle to more ductile [19]. These properties are particularly significant in applications that require both strength and flexibility. Furthermore, the presence of PHBV enhances the elongation at break and fracture toughness of the compounds, which is crucial in the context of applications requiring impact resistance [20].

The vibration welding process capitalizes on these beneficial properties by ensuring that the welds maintain the output parameters of the materials. Concurrently, the different melting points of PLA and PHBV resulting from their immiscibility can introduce complexities into the welding process [21]. However, vibration welding through local heating and mechanical mixing can mitigate these difficulties, promoting improved interfacial bonding and reducing the risk of phase separation. It is also essential to consider the effect of PHBV crystallization, which can significantly influence the final properties of the mixture [22,23].

In conclusion, the vibration welding technique offers an efficacious approach to enhancing the mechanical properties and performance of PLA/PHBV blends. The optimization of welding parameters, considering the distinct characteristics of both polymers, facilitates the production of durable and sustainable materials suitable for diverse applications. Subsequent investigations should concentrate on a comprehensive analysis of welded interfaces and examining the impact of processing conditions on the mechanical and thermal properties of the resultant composites.

The objective of this research is to determine the practicality of fabricating welded for biocomposites comprised of PLA/PHBV blends with various nanocrystalline cellulose concentrations. The development of advanced composites derived from natural and renewable sources is a critical area of industry and science that requires extensive research to hasten their incorporation into the production process across diverse sectors. Not only are mechanical properties crucial, but the ability to bond these materials is also vital for their practical application. Vibratory welding plays a crucial role in joining biocomposites, facilitating the optimization of their mechanical and functional properties. In the context of biopolymers, such as PLA/PHBV blends, vibration welding enhances material flow and mitigates defects at the joint interface. Through localized heating and mechanical agitation, this technique addresses the challenges associated with melting point disparities and component immiscibility, resulting in enhanced interfacial bonding and a diminished risk of phase separation. The application of vibration welding to biocomposites, including those reinforced with nanocrystalline cellulose, facilitates the production of durable and sustainable materials that combine high strength, flexibility, and impact resistance. Concurrently, owing to its simplicity, efficiency, and cost-effectiveness, this technique supports the development of advanced composites of natural origin, thereby enabling broader implementation in industrial applications.

## 2. Materials and Methods

**Figure 1:**
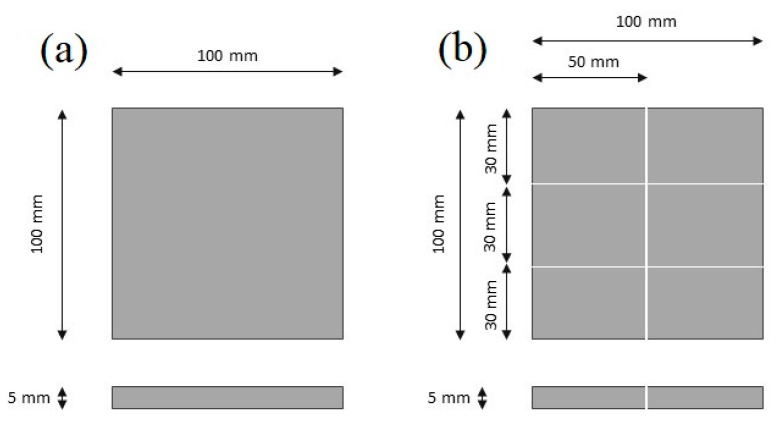
Diagram of the produced samples: (**a**) injection sample diagram; (**b**) diagram of samples prepared for vibration welding.

**Figure 2:**
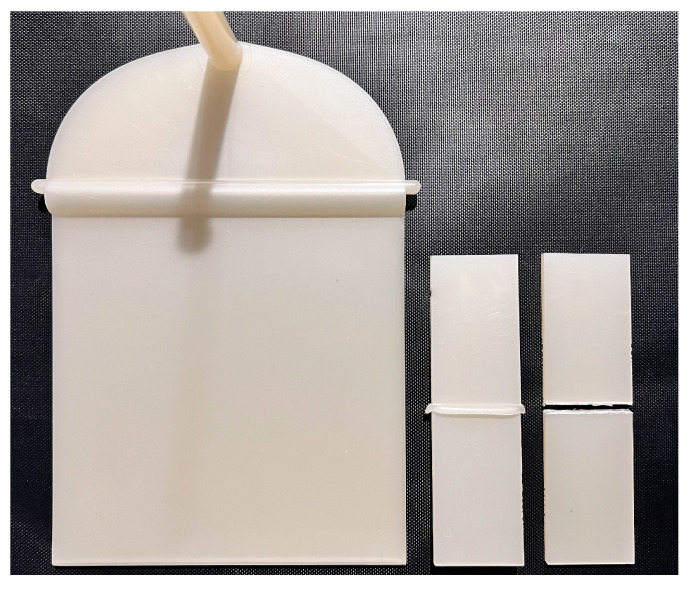
Photograph of injected samples and samples after vibration welding.

The chosen matrix material was a PLA/PHBV polyester blend comprised of 90% PLA and 10% PHBV, supplied by ColorFabb (Belfeld, The Netherlands). Nanocrystalline cellulose (NCC), provided by Nanografi Nano Technology (Jena, Germany), served as the reinforcement material with particle diameters and lengths of 10–20 nm and 300–900 nm, respectively. Polymer composites containing 5, 10, and 15% by weight of NCC were produced. Table 1 presents a description of the manufactured materials. The PLA/PHBV and its composites were fabricated through injection molding. The resulting shapes were parallelograms with dimensions of 100 × 100 × 5 mm (Figure 1a). Subsequently, samples measuring 30 × 50 × 5 mm were cut from these injection-molded materials (Figure 1b) and subjected to vibration welding. Figure 2 presents a photograph of the samples.

The samples were welded at a constant frequency of 240 Hz, which is the constant frequency for the Branson M-112H vibrating welding machine (Branson Ultraschall, Dietzenbach, Germany). The research was conducted in two phases. The initial phase entailed the identification of optimal vibration welding procedures, which included variables such as the vibration amplitude (in millimeters), welding duration (in seconds), and pressure (in atmospheres). Preliminary mechanical tests, including static tensile examination, were performed on the samples. Thereafter, the specimens were prepared for additional testing based on the selected welding parameters. The welding process was carried out using a vibration welding machine. The densities of the materials were measured using a RADWAG WAS 22W laboratory scale (Radwag, Radom, Poland).

The static tensile test was conducted using a Shimadzu AGS-X 10 kN (Shimadzu, Kyoto, Japan) testing machine with a testing speed of 5 mm/min. A series of strength tests were performed using digital image correlation (DIC), a non-contact, image-based method that processes digital images of deforming objects to calculate full-field displacements, deformations, and/or vibrations. DIC provides more comprehensive data than traditional strain gauges and extensometers and offers both local and average information on deformation during mechanical testing. The GOM Aramis SRX system (Zeiss, Braunschweig, Germany) and GOM Corelate Pro version 2022.0.7.157889 were employed for DIC measurements (Zeiss, Braunschweig, Germany). The results of the mechanical tests were subsequently compared with those of the corresponding mathematical models.

The initial mechanical hysteresis loops were designed to demonstrate the viscoelastic properties of the fabricated composites. The experiment was conducted using an applied load of up to 60% of the maximum braking force identified during the tensile test. The purpose of this method was to evaluate the displacement that occurs during repetitive use and the energy dissipation. The tests were performed using a Shimadzu AGS-X 10 kN testing machine (Shimadzu, Kyoto, Japan) equipped with software for energy dispersion analysis (Autograph Trapezium X). The loading and unloading speed was 10 mm/min. The degradation test was carried out using a QUV ACCELERATED WEATHERING TESTER aging chamber (Q-LAB Corporation, Westlake, OH, USA). The samples were placed in an aging chamber, and then their current condition was checked every 7 days. The experiment replicates the effects of environmental exposure, specifically sunlight and rainfall, that materials might experience over extended periods ranging from months to years under outdoor conditions. Table 2 outlines the specifications for a single cycle of the weathering process, which should be conducted continuously for 1000 h according to the ASTM G154-23 [24].

The microstructure observations were conducted on a scanning electron microscope JEOL JSN5510LV (JEOL Ltd., Tokyo, Japan). Prior to testing, the sample surface was coated with a conductive gold layer using a JEOL JEE-4X vacuum evaporator (JEOL Ltd., Tokyo, Japan).

## 3. Results and Discussion

### 3.1. Preliminary Tests

Vibration welding is a commonly employed technique for joining thermoplastic composites, effectively producing robust joints through the generation of frictional heat. The thickness of the melt layer and temperature profiles are critical factors that influence the microstructure of the weld zone. It is essential to optimize the welding parameters to achieve strong and dependable welds. By comprehending the impact of the key factors, the welding process can be controlled, and the desired mechanical properties of the joints can be ensured. [13,25,26]. The data presented in Table 3 include the initial welding parameters, the resulting density of the composite materials, and the maximum force necessary to separate the joint. The selection of these parameters was based on a visual examination of the material behavior during the welding process. The study results did not exhibit a clear pattern. Different effects of the parameters were observed for each of the tested composites. For the polymer blend, the highest force required to break the joint was observed for the material welded with a higher amplitude and average process duration. For the materials modified with nanocrystalline cellulose, the timing and amplitude of the vibrations affected the strength of the joint.

Vibration amplitude is considered a vital factor in the vibration welding of polymer composites, as it has been shown to exert a noteworthy influence on parameters such as power consumption, energy utilization, shear strain velocity, and plastic deformation [27]. Elevating the amplitude of vibration results in a higher consumption of energy and a stronger weld [28]. A greater amplitude leads to a more rapid dissipation of frictional energy, resulting in an increase in the temperature surrounding the weld joint and enhancing the capacity for shear plastic deformation [29]. Furthermore, it influences the structure of the weld at the microscopic level and the mechanical properties of the material. Specifically, when a high amplitude is combined with a low pressure, it results in a wider heat-affected zone, reduced crystal orientation, birefringence, and pronounced recrystallization zones [25]. The amplitude of vibration plays a significant role in the distribution and absorption of energy and stress within the welded components during ultrasonic welding processes [30]. The outcomes of research studies demonstrate that the length of time for vibration welding has a substantial impact on both the process and the characteristics of the weld. Patham and Foss demonstrated that welding time or penetration distance has an impact on the microstructure development in the welding zone and the weld strength of thermoplastics [13]. Tucker et al. emphasized the importance of setting the holding time during vibration welding to half the vibration time in industrial practice to ensure optimal joint strength [31]. In summary, welding time has a significant impact on the microstructure, mechanical properties, and overall quality of the joint in the vibration welding of polymer composites. It is essential to control and optimize the welding time to achieve the desired strength and integrity of the welded components. In the vibratory welding of polymer composites, weld pressure is a crucial factor in determining joint strength and weld quality. Research on Nylon 66 vibration welding has demonstrated that lower pressure results in higher joint strength [32]. Upon examination of the crack surfaces in long glass-fiber-reinforced composites, it was observed that low pressure can lead to a lack of properly reoriented fibers, ultimately impacting the strength of the weld [13]. Research on thermoplastic matrix composites has demonstrated that the application of welding pressure and management of weld depth can regulate the shear strength of overlapping regions in polypropylene and polyamide-12 composites reinforced with glass fabric [14]. The size of the weld area diminishes as the pressure increases, as evidenced by the vibratory welding of hybrid polyoxymethylene/multilayer carbon nanotubes [33]. The importance of weld pressure in relation to the effects of amplitude, melt, and pressure on the tensile strength of unreinforced and glass-fiber-reinforced nylon 6 and 66 cannot be overstated [34].

### 3.2. Structure Analysis

Based on preliminary tests for the analysis of the produced joints, welding parameters were selected for the specimens exhibiting optimal mechanical properties. The parameters utilized are presented in Table 4.

**Figure 3:**
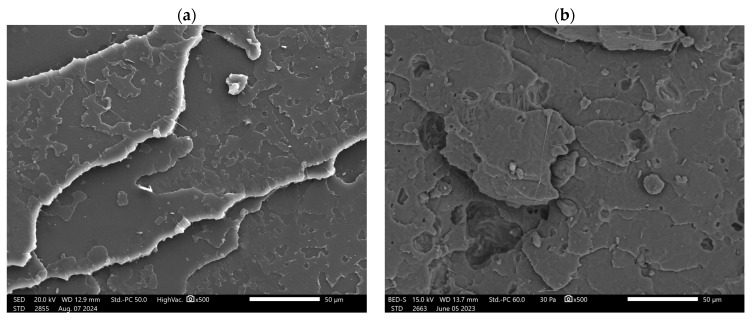

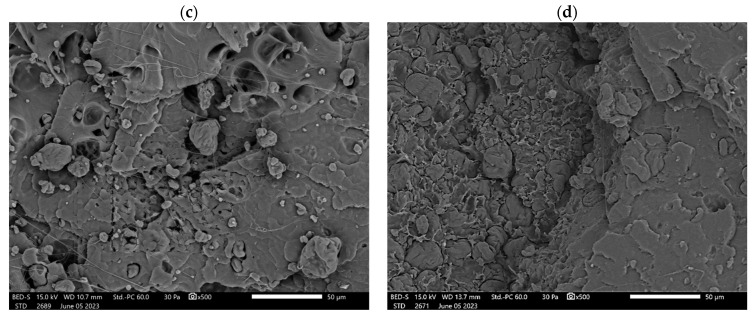
Microstructure of manufactured composites: (**a**) PLA/PHBV; (**b**) 5%NCC; (**c**) 10%NCC; (**d**) 15%NCC.

The initial phase of the research involved analyzing the distribution of reinforcement within the material matrix. Figure 3 presents images of the composite structures. Analysis of these images revealed a lack of uniformity in the distribution of the reinforcement within the matrix and the formation of nanocellulose agglomerates in the composite. The degree of unevenness in the reinforcement distribution within the matrix increased proportionally with the content of nanocrystalline cellulose. This particular arrangement is attributed to the non-uniform flow of the material during the injection process and the potential moisture content in the natural filler. During the flow of material in the injection mold, the fibers orient themselves in the direction of the molten mass flow. Consequently, the arrangement of the fibers is correlated with the geometry of the mold [35]. Cellulose agglomeration in polymer composites is a significant problem that can significantly affect the performance and properties of these materials. Cellulose fibers tend to form agglomerates for various reasons, such as the presence of hydroxyl groups on the surface, hydrogen bonding, and the hydrophilic nature of cellulose. Such agglomerates can act as stress concentration points in the composite, leading to a reduction in the impact resistance, strength properties, and thermal stability [36,37,38].

### 3.3. Mechanical Analysis

**Figure 4:**
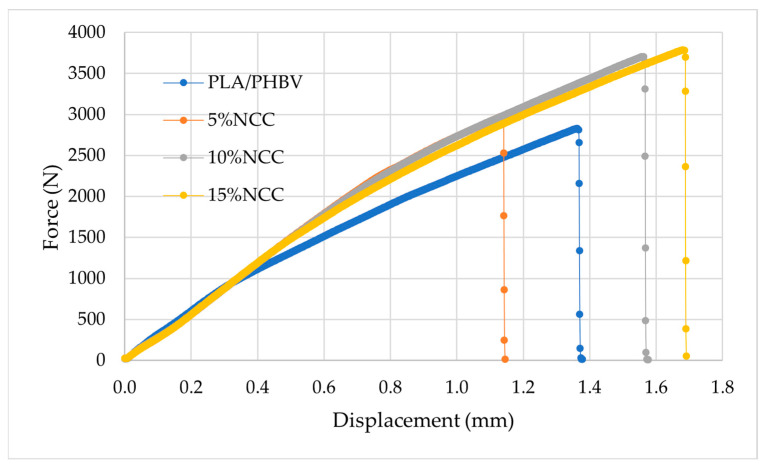
Example tension curves for the welded specimens.

The produced samples were then subjected to strength tests. Representative force-displacement curves for the welded specimens obtained during the static tensile test are presented in Figure 4.

The experimental results demonstrated that the fabricated joints exhibited superior strength compared to the base material. As the filler content increases, the strength of the junction increases, reaching its maximum value for composites with the addition of 15% nanocrystalline cellulose. The enhanced joint strength in the composites is associated with the vibration welding process itself, which affects the polymer structure through melting and reassembly. This process may contribute to increased adhesion between components, an increased number of grafts at the interface, and alterations in the nature of the structure itself. However, it is crucial to consider the appropriate process parameters. Research findings indicate that the incorporation of NCC into diverse polymer matrices substantially enhances the tensile strength, elastic modulus, and overall mechanical properties, attributable to its distinctive structure and extensive specific surface area. A principal mechanism of action of NCC is the enhancement of the interfacial bond between the matrix and the reinforcing phase. For instance, robust hydrogen bond interactions between NCCs and polymer matrices such as nylon 6 or polycaprolactone result in a significant improvement in mechanical properties [39,40]

In addition to strengthening the interfacial bonds, NCC plays a vital role in inhibiting the propagation of cracks in the composite material. The high aspect ratio and reactive surface of NCC facilitate efficient stress distribution, which reduces the stress concentration at the crack termini, thereby minimizing the risk of further propagation [41]. Studies have demonstrated that NCC-reinforced composites exhibit enhanced thermal stability and improved mechanical properties, which are attributed to the uniform dispersion of NCC in the polymer matrix and improved load transfer [42,43]. NCC’s capacity to efficiently dissipate stress and increase mechanical resistance contributes to the longevity and reliability of composite materials.

Furthermore, the incorporation of NCC is associated with improved thermal stability, which is crucial for maintaining mechanical integrity under varying temperature conditions. The presence of NCCs increases the activation energy required for the movement of polymer chains, indicating a higher thermal resistance and stability of the composites [42]. This property is particularly significant in applications that require resistance to thermal and UV degradation, which further enhances the reliability and durability of composite joints.

To explain the structural changes in vibration-welded polymer composite joints, phenomena such as friction, melting, deformation, melt flow under pressure, solidification, and microstructure development must be considered, which affect the strength of the joint [25]. This technique also allows the joining of incompatible polymers by means of an intermediate layer [42] and the generation of bonds in non-sticky polymers by the oscillatory movement of the elements [43]. Understanding these factors is crucial for optimizing the process and ensuring the strength of the joints. Vibration welding is a versatile method that allows the joining of amorphous and semi-crystalline polymers, offering a wide range of possibilities for polymer composites [44].

The produced joints were also subjected to tensile testing using digital image correlation. Digital Image Correlation (DIC) is extensively employed in the structural analysis of polymer composites. DIC has been utilized to detect delamination in polymer composites, providing information on the internal damage to structures [45,46]. In the study of cellulose composites, DIC was employed to analyze the mechanical behavior and deformation distribution in the composites. Zhang et al. investigated the reinforcing effect of nanocrystalline cellulose on the mechanical and thermal properties of poly (lactic acid) composites, emphasizing the significance of cellulose additives as a reinforcing filler [45]. Aravind et al. examined advances in the use of nanocrystalline cellulose to reinforce natural fibers in biocomposites, highlighting developments in the field [47]. Furthermore, digital image correlation was applied to the simulation of hybrid carbon-aramid composite materials, demonstrating its efficacy in the mechanical characterization and analysis of composite structures [48].

**Figure 5:**
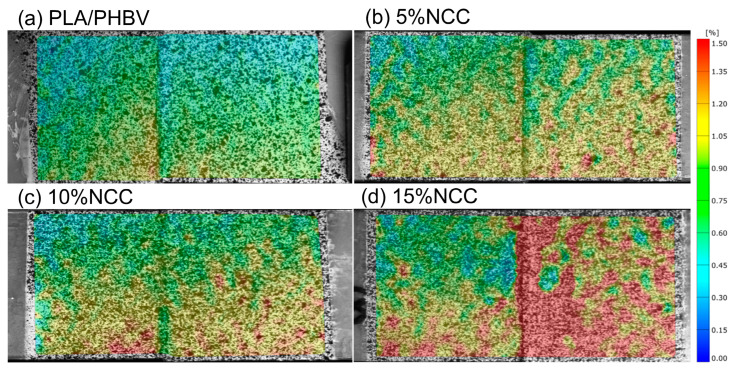
Examples of DIC deformation maps for the tested joined samples.

**Figure 6:**
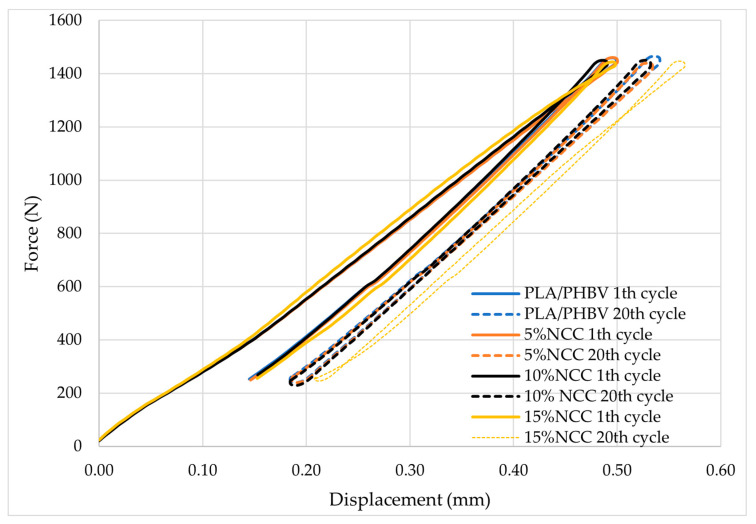
First and twentieth hysteresis loops for welded PLA/PHBV composites.

Sample images recorded using the digital image correlation (DIC) technique illustrate the regions where deformations occurred in the tested specimens. Figure 5, obtained through the DIC technique, depicts the deformation distributions in the y-direction, corresponding to the direction of the tension force. The results are represented by a color-coded diagram on a vertical scale, reflecting the range from the minimum to maximum strain values. Furthermore, the illustrations denote the moments immediately preceding the fracture of the composites during the experiments. The areas exhibiting the highest deformation values in the y-direction are marked by the red regions along the length of the measurement. Numerous red regions were observed in the images, which were heterogeneously distributed on the surface. The results of the test correlated with those of the static tensile test. As the nanocrystalline cellulose content increased, the area with red regions expanded, indicating an increase in the local deformation. The images presented in Figure 6 were recorded at a deformation rate of 1 mm/min.

### 3.4. Energy Dissipation

Polymer composites are extensively utilized in various industries due to their energy absorption capabilities. Energy dissipation in polymer composites is a critical aspect that significantly influences their performance in applications such as impact resistance and crash resistance. Research has demonstrated that factors such as the type, orientation, and volume fraction of fibers play a crucial role in determining the energy absorption properties of polymer composites. For instance, the incorporation of natural fibers, such as basalt, flax, or kenaf, has been demonstrated to enhance energy absorption capabilities [49,50,51]. Furthermore, aligning fibers, such as carbon fibers, parallel to the direction of the load can improve energy absorption during impacts [51]. Comprehending the energy absorption characteristics of polymer composites is essential for optimizing their performance in various applications. Through the study and manipulation of parameters such as fiber volume fraction, orientation, and composite structure, researchers can tailor polymer composites to exhibit superior energy dissipation properties, rendering them suitable for demanding applications that require high impact resistance and energy absorption capacity.

**Figure 7:**
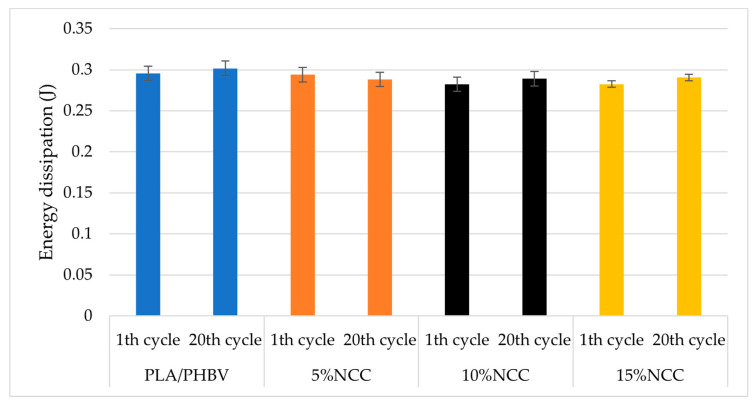
Differences in energy dissipation between the first and twentieth load cycles.

The composite samples were subjected to mechanical hysteresis loop tests involving a low-cycle load, which enabled the determination of the energy dissipated in the material during cyclic load changes (Figure 6 and Figure 7). In unreinforced materials, energy dissipates primarily due to internal friction between molecules, resulting in the generation of thermal energy. This method can also be used to analyze the interactions between molecules in composite materials. According to the theory of Cieszyński and Topolinski [52], studies conducted on multiphase materials have demonstrated that the initial load cycles result in the elimination of local stresses and material defects. The loading and unloading processes function as relaxation processes, leading to cracks in the interfacial areas that have been subjected to maximum stress. Consequently, after several cycles, the dissipation energy stabilizes, with its value remaining at an almost constant level for the subsequent several dozen cycles [53,54,55].

Energy dissipation in polymer composites is a critical factor in elucidating their mechanical behavior and performance. This phenomenon is frequently associated with hysteresis loops, which represent the energy lost during charge and discharge cycles. Several studies have examined the relationship between energy dissipation and the mechanical properties of polymer composites. For example, Nakai and Yokoyama [56] demonstrated that the area encompassed by the stress-strain hysteresis loop, which represents the dissipation energy, increases with increasing strain rate in various polymers tested, indicating internal dynamic viscoelasticity and pronounced elastic effect post-discharge. Li et al. [57] investigated the influence of interfacial bonding properties on the cyclic tensile behavior of composite materials and observed that the energy dissipated through hysteresis increases at low peak stress, corresponding to interface failure.

Based on the aforementioned theories, research findings of other scientists, and results of the static tensile test of conventional strength specimens, it can be concluded that the switches exhibit stability in their properties under low-cycle loads. The value of the scattering energy remained relatively constant during the initial twenty cycles, and these investigations corroborated the observations recorded in the static tensile test, which demonstrated that the material modified with the 15% addition of nanocellulose exhibited the highest deformation value compared to the other composites. These findings suggest that the joining parameters were appropriately selected.

### 3.5. Degradation Tests

Accelerated aging tests were carried out on universal strength samples. The study included cyclic changes in UV radiation, temperature, and water spray. The results of this study are presented in Table 5.

For the PLA/PHBV samples, the effect of aging was the greatest because after over 300 h of the aging tests, the samples were completely degraded, which prevented the performance of strength tests. After removal from the aging chamber, the samples became brittle. However, strength tests were performed on composites with the addition of nanocellulose, and the results indicated that the presence of NCC increased the resistance of the materials to the aging processes. The strength tests showed a significant effect of the aging parameters on the mechanical properties, causing a decrease in the tensile strength and Young’s modulus. These results were obtained after over 300 h of accelerated aging. Longer aging times led to the complete degradation of the materials, except for the composite with 15% wt. NCC retained its durability for 660 h, but its strength properties dropped dramatically.

Nanocrystalline cellulose is garnering significant attention in scientific research, particularly due to its potential to enhance the mechanical properties of biodegradable polymer matrices. The incorporation of NCC into such materials can substantially augment the tensile strength and elastic modulus, as demonstrated by studies on polymers such as polylactic acid and poly (butylene succinate) (PBS) [58,59]. Specifically, the incorporation of NCC into PBS results in a substantial enhancement of both the tensile strength and elongation at break, which can be attributed to the homogeneous distribution of nanofillers and the robust interfacial adhesion between NCC and the polymer matrix [59]. The mechanical reinforcement provided by NCC is attributed to its high aspect ratio and crystallinity, which facilitate efficient load transfer within the polymer matrix [42,60].

The incorporation of NCCs into biodegradable polymers significantly influences the biodegradation processes. Research has demonstrated that NCCs enhance water absorption, thereby facilitating the hydrolytic degradation of the polymer matrix and promoting polymer chain disintegration. For instance, in PLA composites, NCCs improve water penetration, thus accelerating polymer hydrolysis [61]. Nevertheless, it is crucial to acknowledge that an excessive nanocrystalline cellulose content may potentially diminish the biodegradability of the material due to the formation of strong hydrogen bonds between NCCs and the polymer, which could impede the access of microorganisms to the matrix [62].

Researchers point out that the accelerated aging technique employed in this study to mimic long-term environmental impacts does not perfectly replicate natural aging conditions. While accelerated aging tests intensify factors like temperature, humidity, or UV radiation to speed up degradation processes, they fail to capture the full complexity and variability of natural environments. Consequently, findings from such tests may not align with observations in real-world scenarios, necessitating careful interpretation and application of the results to practical situations. Emphasizing this distinction is essential for a comprehensive understanding of how materials behave throughout their actual lifecycle.

## 4. Conclusions

This investigation aimed to assess the viability of creating vibratory welded joints using composite materials derived from polylactide blends and polyhydroxyalkanoate modified with nanocrystalline cellulose. This study incorporated cellulose concentrations of 5, 10, and 15 wt%. The specimens were fabricated through injection molding and subsequently joined via welding techniques. The research commenced with an examination and refinement of the welding parameters, along with an evaluation of the composite structure. Notably, the composites exhibited heterogeneous reinforcement distributions characterized by nanocrystalline cellulose agglomerations. Subsequent strength testing of the welded joints revealed the beneficial impact of nanocrystalline cellulose incorporation. Composites containing this additive demonstrated superior joint strength compared to the base material, with a peak strength observed at 15% cellulose content. In the case of vibration-welded specimens, the welding process was found to enhance interfacial adhesion, thereby contributing to increased joint strength. DIC studies facilitated the analysis of deformations in the samples, indicating that a higher nanocellulose content led to greater local deformation, thus reducing brittle fracture. High strain values were localized in areas with agglomerates, correlating with the results of the static tensile tests. The joints were also subjected to mechanical hysteresis loop tests, which revealed that the nanocellulose composites exhibited stable properties under low-cycle loads, indicating satisfactory resistance of the material to varying loads. The energy dissipated during cyclic load changes remained constant, suggesting sufficient adhesion between the components and the absence of grain detachment from the matrix during cyclic loads in the initial few dozen cycles. In conclusion, this research has demonstrated that the incorporation of nanocrystalline cellulose into PLA/PHBV blends can positively influence the strength properties of composite materials. Furthermore, these materials can be successfully joined using a vibration welding technique and appropriate parameter selection.

## Figures and Tables

**Table 1:** Description of the materials produced.

Designation	Description	Density, (g/cm^3^)
PLA/PHBV	PLA/PHBV blend containing 90% PLA and 10% PHBV as the base material	1.25
5%NCC	PLA/PHBV blend with 5 wt% NCC	1.26
10%NCC	PLA/PHBV blend with 10 wt% NCC	1.27
15%NCC	PLA/PHBV blend with 15 wt% NCC	1.28

**Table 2:** Characteristics of the aging cycle.

Cycle	Function	Intensity (W/m^2^/nm)	Temp. (°C)	Time (min)
	UV light	1.55	60	8:00
	Water spraying	-	-	0:15
	Condensation	-	50	3:45

**Table 3:** Initial parameters of vibration welding.

Index	Vibration Welding Parameters			Max Force, (N)
	Vibration Amplitude a, (mm)	Welding Time t_z_, (s)	Pressure p, (atm)	
PLA/PHBV_1	1.0	6.5	1.0	640 ± 258
PLA/PHBV_2	1.0	3.0	1.0	2874 ± 125
PLA/PHBV_3	1.2	4.5	1.0	2157 ± 352
5%NNC_1	1.0	6.5	1.0	2011 ± 147
5%NNC_2	1.0	4.5	1.0	1590 ± 156
5%NNC_3	1.6	3.0	1.0	2894 ± 65
10%NNC_1	1.0	5.0	1.5	1552 ± 24
10%NNC_2	1.2	4.0	1.5	3030 ± 48
10%NNC_3	1.6	5.0	1.0	3705 ± 25
15%NNC_1	1.2	4.5	1.0	2357 ± 158
15%NNC_2	1.6	3.0	1.0	2830 ± 235
15%NNC_3	1.6	5.0	1.5	3787 ± 46

**Table 4:** Welding parameters of the manufactured composites.

Index	Vibration Welding Parameters			Max Force, (N)
	Vibration Amplitude a, (mm)	Welding Time t_z_, (s)	Pressure p, (atm)	
PLA/PHBV	1.0	3.0	1.0	2874 ± 125
5%NNC	1.6	3.0	1.0	2894 ± 65
10%NNC	1.6	5.0	1.0	3705 ± 25
15%NNC	1.6	5.0	1.5	3787 ± 46

**Table 5:** Strength properties before and after the accelerated thermal aging process lasting 330 h.

Material	Condition	Young’s Modulus (MPa)	Tensile Strength (MPa)
PLA/PHBV	conditioned	4899 ± 119	61.1 ± 1.1
	aged	-	-
5%NNC	conditioned	7319 ± 846	63.4 ± 2.4
	aged	3265 ± 987	9.3 ± 1.3
10%NNC	conditioned	8777 ± 622	63.7 ± 3.6
	aged	4786 ± 1223	13.0 ± 3.4
15%NNC	conditioned	9097 ± 580	64.8 ± 5.1
	aged	4947 ± 1233	14.7 ± 3.7
	aged *	1136 ± 852	4.7 ± 2.3

* Strength properties after 660 h.

## Data Availability

The raw data supporting the conclusions of this article will be made available by the authors upon request. The data are not publicly available due to [University does not provide a public open repository].
